# POU2AF1 promotes MSCs adipogenesis by inhibiting HDAC1 expression

**DOI:** 10.1080/21623945.2021.1918863

**Published:** 2021-05-05

**Authors:** Yaqing Wang, Luyang Wang, Zhongping Su, Wei Sun, Mi Zhang, Chuanxi Yang, Jingxin Zhou, Li Jiang, Xiangqing Kong

**Affiliations:** a Department of Cardiology, Geriatric Hospital of Nanjing Medical University, Nanjing, Jiangsu, China; b Department of Cardiology, The First Affiliated Hospital of Nanjing Medical University, Nanjing, Jiangsu, China; c Department of Biotherapy, Second Affiliated Hospital, Nanjing Medical University, Nanjing, Jiangsu, China; d Department of Gynecological Tumor, Nanjing Maternity and Child Health Hospital, Nanjing, Jiangsu, China; e Department of Cardiovascular Surgery, The First Affiliated Hospital of Nanjing Medical University, Nanjing, Jiangsu, China; f Department of Pediatrics, The First Affiliated Hospital of Nanjing Medical University, Nanjing, Jiangsu, China

**Keywords:** POU2AF1, mesenchymal stem cells, hdac1, adipogenesis, overweight

## Abstract

Excessive production of visceral adipose is a major risk factor of many diseases. Inhibiting the adipogenesis of mesenchymal stem cells (MSCs) will be an efficient way to block adipose production. We illuminated POU class 2 homeobox associating factor 1 (POU2AF1) may promote MSCs adipogenesis by histone deacetylases 1 (HDAC1) signalling. Human retroperitoneal adipose-derived mesenchymal stem cells were isolated from overweight and control groups of patients. IncRNA microarray was used to identified gene expression levels. Adenovirus transduction and cellular small-interfering RNA transfection were used to achieve overexpression and interference of POU2AF1 or HDAC1. Adipogenesis was identified by Oil-red O staining, triglycende, cholesterol assay, real-time PCR and Western Blot. POU2AF1 expression was upregulated in retroperitoneal adipose tissue of overweight patients, and increased during adipogenesis. Overexpression of POU2AF1 promoted spontaneous adipogenesis without adipogenic treatment. Silencing of endogenous POU2AF1 in MSCs inhibited adipogenesis. Overexpression of POU2AF1 alleviated the translocation of HDAC1 to the nucleus. The mRNA level of HDAC1 was also reduced. Co-transfection of Ad-POU2AF1 and Ad-HDAC1 partially reversed the promotion effect of POU2AF1 overexpression in MSCs spontaneous adipogenic differentiation. POU2AF1 involves in the natural differentiation of human mesenchymal stem cells. Overexpression or silencing POU2AF1 could effectively induce or inhibit the adipogenesis by HDAC1 signaling.

## Introduction

Overweight and obesity are considered as one of the major risk factors for many cardiovascular diseases. They have direct and indirect effects on worsening arterial hypertension, atherosclerosis, and related metabolic syndrome, including insulin resistance and type 2 diabetes [1–3]. Intra-abdominal adipose tissue depot, which is mainly made up of visceral intraperitoneal fat, primarily omental and mesenteric fat, mainly contributes to obesity and is strongly associated with cardiovascular risk factors [4,5]. The major white adipose tissue (WAT) depots in the body are found in the visceral cavity and subcutaneously, but these effects of pathologic inflammation and insulin resistance are much more pronounced in the accumulation of visceral adipose capacity [6].

Adipogenesis is the process by which mesenchymal stem cells (MSCs) differentiate into adipocytes. MSCs are capable of self-renewal and differentiation into mesenchymal cell types, including adipocytes, osteoblasts, chondrocytes and myoblasts, both in vitro and in vivo [7]. As we known, adipose tissue increases in two main ways: hypertrophy (enlargement of existing adipocytes) or hyperplasia (formation of new adipocytes through differentiation of resident precursors) [8]. As reported, white adipocyte numbers increase through adolescence but remain stable in adulthood. However, adipogenesis takes place in adults since adipocytes undergo nearly a 10% annual turnover in human adult and short-term overfeeding increases adipocyte cell numbers [9].

Adipocyte differentiation is a regulated process driven by transcription cascade mediated by a battery of transcription factors, including CCAAT/enhancer-binding proteins (C/EBPs) and peroxisome proliferator-activating receptor (PPAR) γ, of which targets genes are responsible for lipid metabolism. They increase lipid accumulation and lead to the development of a mature lipid laden adipocyte [10]. Peroxisome proliferator-activated receptor-γ (PPAR-γ) which belongs to the pleiotropic nuclear receptor 1 C (NR1C) family, and C/EBP-α which belongs to the C/EBP family of transcription factors, have been shown to have an important role in the initiation of adipogenesis both in vivo and vitro. In vitro, mutant PPAR-γ, altering cysteine residues at positions 156 and 159 to serine, is unable to stimulate adipose differentiation [11]. In vivo, PPAR-γ FKO mice showed severe lipoatrophy [12]. C/EBP-α-/- mice failed to store lipid in vivo [13]. Furthermore, PPAR-γ and C/EBP-α are required for maintaining mature adipocyte function. Histone acetylation plays an important role during adipogenesis, but the role of the regulators remains unclear [9].

Histone acetylation and deacetylation are regulated by the balance action of histone acetyltransferases and histone deacetylases (HDACs). HDACs consist of four major classes: class I (HDAC1, −2, −3, and −8), class II (HDAC4, −5, −6, −7, −9, and −10), class III (SIRT1 to −7), and class IV (HDAC11). HDAC1 acts to remove acetyl groups from lysine residues in histone and cellular proteins to regulate gene expression and protein activity [14]. HDAC1 has been reported to regulate the early steps of adipocyte differentiation [15], and it could be recruited to the C/EBP-α promoter targeting for degradation and dislodgement during adipogenesis [16]. A study in 20 showed that the treatment of 3T3-L1 cells with the non-specific HDAC inhibitor trichostatin A (TSA) resulted in ablation of lipid accumulation or expression of mature adipocytes markers [17]. All of these studies above indicate that HDAC1 impacts a significant effect on adipogenesis.

POU2AF1 (POU class 2 homeobox associating factor 1), also known as BOB1, OBF-1 or OCAB, was a B-cell-specific transcriptional co-activator protein and was believed to facilitate B-cell-specific Ig expression. POU2AF1 normally interacts with Oct-1 and Oct-2 proteins and increases the binding affinity for DNA [18]. However, it remains unknown whether and if so how POU2AF1 may regulate MSCs adipogenesis.

It has been rarely reported the interaction between POU2AF1 and HDAC1. However, based on previous research, we assumed that POU2AF1 may possibly has a role in the adipogenesis of MSCs via HDAC1.

Based on these questions, our study focuses on hyperplasia in which we investigated the role of POU2AF1 in MSCs adipogenesis. mRNA microarray was detected to find some possible mRNAs related to adipogenesis. Based on what our team has found that POU2AF1 was upregulated in retroperitoneal adipose tissue from overweight people, it is assumed that POU2AF1 plays an important role in adipogenesis. Then, we found POU2AF1 is gradually upregulated during MSCs adipogenesis and promotes spontaneous adipogenic differentiation without adipogenic treatment. As expected, silencing endogenous POU2AF1 inhibits adipogenesis. Meanwhile, overexpressing HDAC1 rescues the inhibition effect of silencing POU2AF1 in MSCs. Our study revealed that POU2AF1 promotes MSCs adipogenesis by inhibiting HDAC1 expression.

## Methods

### Human subjects

Human retroperitoneal adipose tissues were obtained from patients who underwent radical nephrectomy for kidney cancer. A total of 60 retroperitoneal adipose tissue samples from participants were collected, while 25 of the human retroperitoneal adipose tissues were from overweight group (BMI >25 kg/m^2^) [19] and 35 of the tissues were from control group (BMI≦25 kg/m^2^). Half of each sample specimen was used to prepare human retroperitoneal adipose-derived MSCs whereas the other half was immediately stored for RNA test later (Life Technologies). This study was approved by the Ethical Committee of the Jiangsu Province Hospital (First Affiliated Hospital of Nanjing Medical University) and written informed consent was obtained from each patient.

### Human retroperitoneal adipose-derived mesenchymal stem cell isolation and culture

Human retroperitoneal adipose-derived MCSs were isolated by collagenase digestion as described previously [20] and cultured in mesenchymal stem cell medium (MSCM; Cat. 7501, ScienCell) (Supplemental Figure 1a). All experiments were performed with human MSCs (hMSCs)at passages 3 to 5.

### mRNA microarray

Levels of mRNA between overweight group (n = 5) and control group (n = 5) were analysed by mRNA microarray (Shanghai Biotechnology Corporation). Patients from 2 groups for microarray were strictly selected. A one-to-one matches pattern was used to pick up enrolled patients and there were no differences between each two patients in baseline data, family history, tumour type and pathological staging, and preoperative medication. 6 mRNAs were validated in a total of 60 samples using quantitative reverse transcription-polymerase chain reaction. Gene expression levels were normalized with β-actin, and data were analysed with StepOne software v2.1 (Applied BioSystems, CA, USA).

### Adenovirus transduction

Human retroperitoneal adipose-derived mesenchymal stem cells were infected with recombinant adenovirus expressing human POU2AF1 or HDAC1 (Genchem, China) according to manufacturer’s instruction. The concentration of adenovirus POU2AF1 was 2.4 × 107PFU/ml and the concentration of adenovirus HDAC1 was also 2.4 × 107PFU/ml.

### Cellular small-interfering RNA transfection

Human retroperitoneal adipose-derived mesenchymal stem cells were transfected with control small-interfering RNA (siRNA) or POU2AF1 siRNA duplex (RiboBio) accordingly with riboFECTTM CP (RiboBio).

### Adipogenic differentiation

For adipogenic differentiation, confluent cells (70–80% confluence) were cultured in differentiation medium (Cat. C-39,436, Promocell). The medium was changed every 2 days.

### Oil-red O staining

After five days adipogenic treatment and five-days normal culture, differentiated adipocytes were gently washed twice with PBS and fixed in 4% paraformaldehyde for 30 min followed by washing. Then stained with 3% (w/v) oil-red O in 60% isopropanol for 60 min, followed by washing with PBS twice. Images of stained cells were obtained under a microscope (Zeiss). For oil-red O quantification, isopropanol was added to each well and optical density was read at 520 nm using a Synergy™ 2 Microplate Reader (BioTek).

### Triglyceride and total cholesterol assay

Triglyceride and total cholesterol content was measured using triglyceride assay kit (A110-1, Nanjing Jiancheng Bioengineering Institute) and total cholesterol assay kit (A111-1, Nanjing Jiancheng Bioengineering Institute) according to manufacturer’s instruction. Briefly, 2% Triton X-100 was added to each well. After homogenization, 2.5 μl homogenate was transferred to 96-well plate. Working reagent (250 μl) was added to each well and incubated for 10 min at 37°C. The optical density was read at 510 nm using a Synergy™ 2 Microplate Reader (BioTek). Triglyceride and total cholesterol content was normalized to total cellular protein.

### Western blot analysis

Proteins were extracted with M-PER or NE-PER Nuclear and Cytoplasmic Extraction Reagents (Thermo) according to manufacturer’s protocol. Proteins were separated by SDS-PAGE and transferred onto a polyvinylidene difluoride membrane (Millipore). After blocking, membranes were incubated overnight with primary antibody and an HRP-linked secondary antibody (1:5000). The primary antibodies used were anti-POU2AF1 (Cat. PA5-26,105, Thermo Fisher), PPARγ (Cat. 2443, Cell Signalling Technology), C/EBPα (Cat. 8178, Cell Signalling Technology), β-Actin (Cat. T0022, Affinity Biosciences), HDAC1 (Cat. 5356, Cell Signalling Technology), Lamin B1 (Cat. 13,435, Cell Signalling Technology). Image LabTM software was used to quantify band density.

### RNA extraction and Real-time PCR

RNA extraction and Real-time PCR were used both in human sample tests and cell tests. Cell total RNA was extracted using the TRI Reagent® (Cat. T9424, Sigma) and 500ng of RNA was reverse-transcribed into cDNA using PrimeScriptTM RT Master Mix (Cat. RR036A, Takara). Real-time PCR reactions were performed on an ABI Prism 7900 system. The primers of real-time PCR are shown in (Table 1Table 2). The cycle conditions of real-time PCR were 95°C 5 min, followed by 40 cycles of 95°C 10 sec, 60°C 20 sec and 72°C 20 sec.

**Table 1.** Top 20 significantly differentially expressed mRNAs

RNA Accession	Gene Symbol	Gene Description	Trend	Chr	Start	End	P values	Fold change
NM_198440	DERL3	transcript variant 3	↑	chr22	70,864	75,374	0.002903223	4.742280195
NM_020070	IGLL1	immunoglobulin lambda-like polypeptide 1, transcript variant 1	↑	chr22	23,573,125	23,580,308	0.005950264	4.480680084
NM_006235	POU2AF1	POU class 2 associating factor 1	↑	chr11	111,352,255	111,379,432	0.003940599	4.455887846
NM_016459	MZB1	marginal zone B and B1 cell-specific protein	↑	chr5	139,387,567	139,389,916	0.002372311	4.064122641
NM_021724	NR1D1	nuclear receptor subfamily 1, group D, member 1	↓	chr17	40,092,783	40,100,725	0.041939218	3.732792972
NM_016352	CPA4	carboxypeptidase A4, transcript variant 1	↑	chr7	130,293,133	130,324,180	0.049507437	3.533124029
NM_173628	DNAH17	dynein, axonemal, heavy chain 17	↓	chr17	78,423,696	78,577,394	0.049880885	3.433161287
NM_001006946	SDC1	syndecan 1, transcript variant 1	↑	chr2	20,200,796	20,225,433	0.011887534	3.373068958
NM_144646	JCHAIN	immunoglobulin J polypeptide, linker protein for immunoglobulin alpha and mu polypeptides	↑	chr4	70,655,540	70,666,631	0.013167924	3.347051932
NM_178171	GSDMA	gasdermin A	↑	chr17	39,962,972	39,977,766	0.037771207	3.056351794
NM_001271082	NKD2	naked cuticle homolog 2 (Drosophila), transcript variant 2	↑	chr5	27,468	57,319	0.002202588	2.97233262
NM_001134450	TMEM130	transmembrane protein 130, transcript variant 1	↑	chr7	98,846,487	98,870,050	0.000104801	2.94901182
NM_002581	PAPPA	pregnancy-associated plasma protein A, pappalysin 1	↑	chr9	116,153,791	116,402,321	0.024748277	2.777150321
NM_001166247	GRIK2	glutamate receptor, ionotropic, kainate 2, transcript variant 3	↑	chr6	101,398,984	102,070,083	0.035459964	2.774817065
NM_001304787	APCDD1L	adenomatosis polyposis coli down-regulated 1-like, transcript variant 2	↓	chr20	58,459,100	58,514,938	0.013940916	2.699423858
NM_018986	SH3TC1	SH3 domain and tetratricopeptide repeats 1	↑	chr4	8,199,332	8,241,103	0.005981494	2.576313861
NM_016953	PDE11A	phosphodiesterase 11A, transcript variant 4	↓	chr2	177,623,248	178,072,755	0.006189209	2.532494199
NM_001174090	SLC4A11	solute carrier family 4, sodium borate transporter, member 11, transcript variant 1	↓	chr20	3,227,416	3,238,189	0.018479644	2.488003915
NM_001178126	IGLL5	immunoglobulin lambda-like polypeptide 5, transcript variant 1	↑	chr22	22,887,779	22,895,833	0.016746568	2.463067142
NM_001164595	PDZRN4	PDZ domain containing ring finger 4, transcript variant 1	↓	chr12	41,188,447	41,574,590	0.007812865	2.457550992

↑: up-regulated, ↓: down-regulated

**Table 2.** Primers used for qRT-PCR of target genes

Primers	Forward	Reverse
POU2AF1	5ʹ-AGCCTCAGCCAGAAGTACCA-3’	5ʹ-GTGAGCCAGTTCCCAAGGTA-3’
PPARγ	5ʹ-GGGATCAGCTCCGTGGATCT-3’	5ʹ-TGCACTTTGGTACTCTTGAAGTT-3’
C/EBPα	5ʹ-CAAGAACAGCAACGAGTACCG-3’	5ʹ-GTCACTGGTCAACTCCAGCAC-3’
HDAC1	5ʹ-CCGCATGACTCATAATTTGCTG-3’	5ʹ-ATTGGCTTTGTGAGGGCGATA-3’
β-Actin	5ʹ-CATGTACGTTGCTATCCAGGC-3’	5ʹ-CTCCTTAATGTCACGCACGAT-3’

### Statistical analysis

Bioinformatics analysis was performed by the SPSS 22.0 software. Experimental data were processed by the GraphPad Prism 6.0 software. All data were expressed as the mean ± standard error of the mean (SEM) from at least three independent experiments. Comparison was conducted with the Independent *t*-test. *P*-values < 0.05 were considered as statistically significant.

## Results

### POU2AF1 expression was upregulated in retroperitoneal adipose tissue of overweight patients

**Figure 1.**
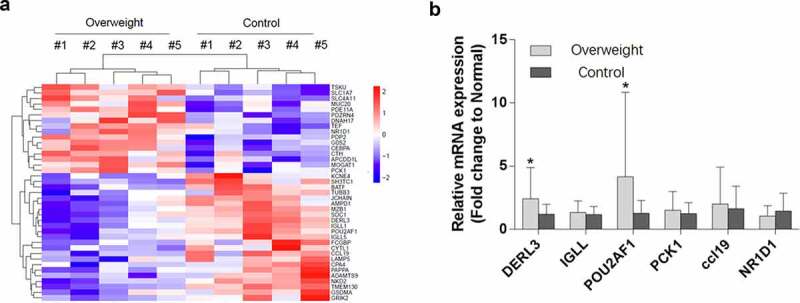
POU2AF1 was highly expressed in human retroperitoneal adipose tissue. (a) Heat map of mRNA microarray from human retroperitoneal adipose of 10 patients for comparison of overweight group (N = 5) and control group (N = 5). (b) POU2AF1 was highly expressed in human retroperitoneal adipose in overweight group versus control group at the RNA level

To determine the key factors for adipose accumulation and overweight, we investigated the global gene expression profiles involved in human overweight. Several genes were upregulated in the retroperitoneal adipose tissue of overweight people, as compared to those of age-matched controls (Table 1 & Figure 1a). In particular, POU2AF1 was significantly up-regulated in overweight people (Table 1&Figure 1a-b).

### POU2AF1 was increased during adipocyte differentiation

**Figure 2.**
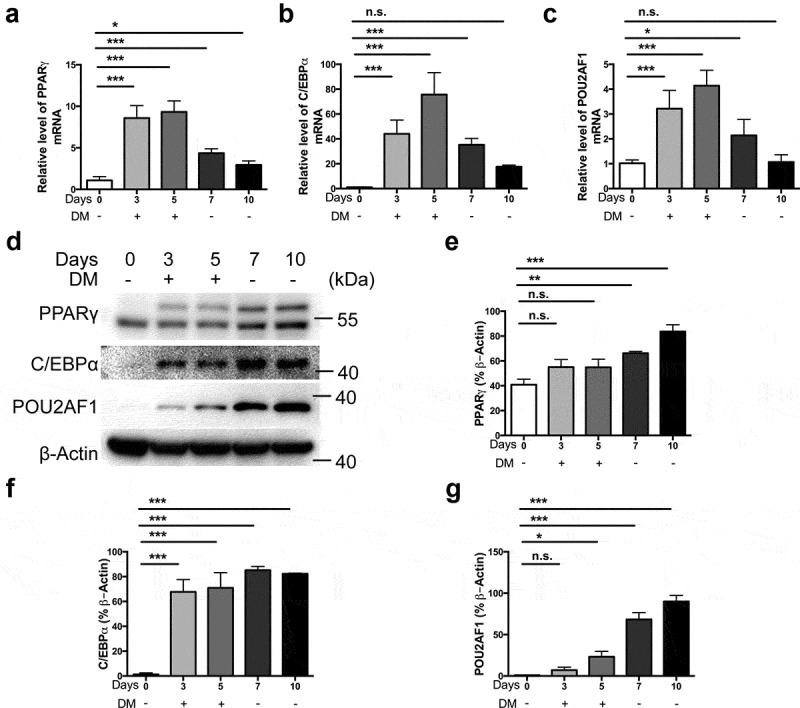
POU2AF1 was increased during adipogenic differentiation. (a) Quantification of PPARγ levels relative to β-Actin during adipogenic differentiation. (b) Quantification of C/EBPα levels relative to β-Actin during adipogenic differentiation. (c) Quantification of POU2AF1 levels relative to β-Actin during adipogenic differentiation. (d) Expression of PPARγ, C/EBPα and POU2AF1 were assessed by western blot. (e) Quantification of PPARγ levels relative to β-Actin. (f) Quantification of C/EBPα levels relative to β-Actin. (g) Quantification of POU2AF1 levels relative to β-Actin. (n = 3 for each experiment. n.s. *P* > 0.05, *P < 0.05, **P < 0.01, ***P < 0.001)

Expression levels of POU2AF1 were examined during adipocyte differentiation of human retroperitoneal adipose-derived mesenchymal stem cells. As expected, PPARγ and C/EBPα expression were gradually increased in hMSCs at day 3 and day 5 after adipogenic treatment (Figure 2a-b). POU2AF1 expression was increased in hMSCs at day 3 and day 5 after adipogenic treatment (Figure 2c). However, the removal of differentiation media decreased the expression of PPARγ and C/EBPα, along with the expression of POU2AF1 at RNA level (Figure 2a-c). Western blot demonstrated that PPARγ and C/EBPα expression were gradually increased in hMSCs after adipogenic treatment and POU2AF1 expression was also increased (Figure 2d-g). The removal of differentiation media did not decrease the protein level of PPARγ, C/EBPα and POU2AF1 (Figure 2d-g). In brief, POU2AF1 expression changed with the expression of PPARγ and C/EBPα, which was the marker of adipogenic differentiation. These results suggest that POU2AF1 has a regulatory role in adipogenesis.

### Overexpression of POU2AF1 promoted spontaneous adipogenic differentiation without adipogenic treatment

**Figure 3.**
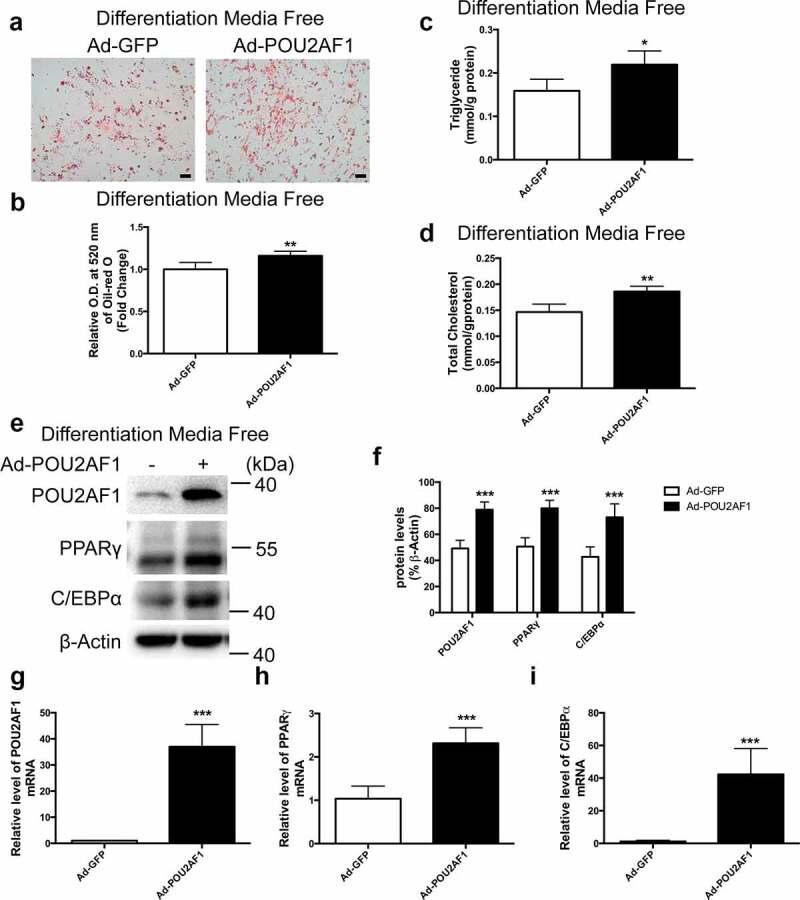
POU2AF1 overexpression promoted hMSCs spontaneous adipogenic differentiation. (a) Neutral lipids formation was determined by oil-red O staining. (b) Oil-red O extracted with isopropanol was measured at O.D. 520 nm. Triglyceride (c) and total cholesterol content (d) were determined in hSMCs without adipogenic treatment. (e) Expression of POU2AF1, PPARγ and C/EBPα were assessed by western blot. (f) Quantification of indicated protein levels relative to β-Actin. (g) Quantification of POU2AF1 levels relative to β-Actin. (h) Quantification of PPARγ levels relative to β-Actin. (i) Quantification of C/EBPα levels relative to β-Actin. (Scale bar: 50 μm. n = 3 for each experiment. *P < 0.05, **P < 0.01, ***P < 0.001)

To determine the effect of overexpression POU2AF1 on adipogenic differentiation, we transduced POU2AF1 adenovirus to hASMCs. Oil-red O staining showed that POU2AF1 overexpression significantly promoted hMSCs spontaneous adipogenic differentiation (Figure 3a-b). Accordingly, the measurement of triglyceride and total cholesterol also indicated that POU2AF1 overexpression promoted hMSCs spontaneous adipogenic differentiation (Figure 3c-d). Protein and mRNA levels of adipogenic transcription factors and marker genes including PPARγ and C/EBPα were increased, along with the overexpression of POU2AF1, compared with Ad-GFP group (Figure 3e-i). Mesenchymal stem cells may differentiate into adipocytes due to cell proliferation to a certain cell density. We have examined the effect of POU2AF1 on cell proliferation, and as shown in Supplemental Figure 1b, inhibited cell proliferation, which suggested that the effect of POU2AF1 on the self-differentiation of MSCs was not realized through proliferation。Then we examined the effect of POU2AF1 on the differentiation of MSCs into adipocytes with the presence of differentiation medium, and the results showed that the promotion effect of POU2AF1 disappeared (S1C-F). The possible reason might be the differentiation medium itself has a strong differentiation promoting effect, which obscured the differentiation promoting of POU2AF1. We then analysed the effect of POU2AF1 on differentiation of MSCs into adipocytes after adding differentiation medium on 0, 3, 5 and 7 days. The results turned out to be that POU2AF1 still up-regulated the expression of PPARγ and C/EBPα on 3^rd^ day, but the up-regulation of C/EBPα disappeared on 5^th^ day, while the up-regulation of PPARγ disappeared on 7^th^ day (S2A-H) . These above further confirm that POU2AF1 only exists effect in early differentiation of MSCs. At the protein level, PPARγ and C/EBPα just stopped increasing after the removal of differentiation medium, while POU2AF1 still kept increasing. However, the increase of POU2AF1 did not cause the increase of PPARγ and C/EBPα, because POU2AF1 only promoted early adipose differentiation, but not happened in late differentiation.

### Silencing of endogenous POU2AF1 in hMSCs inhibited adipogenic differentiation

**Figure 4.**
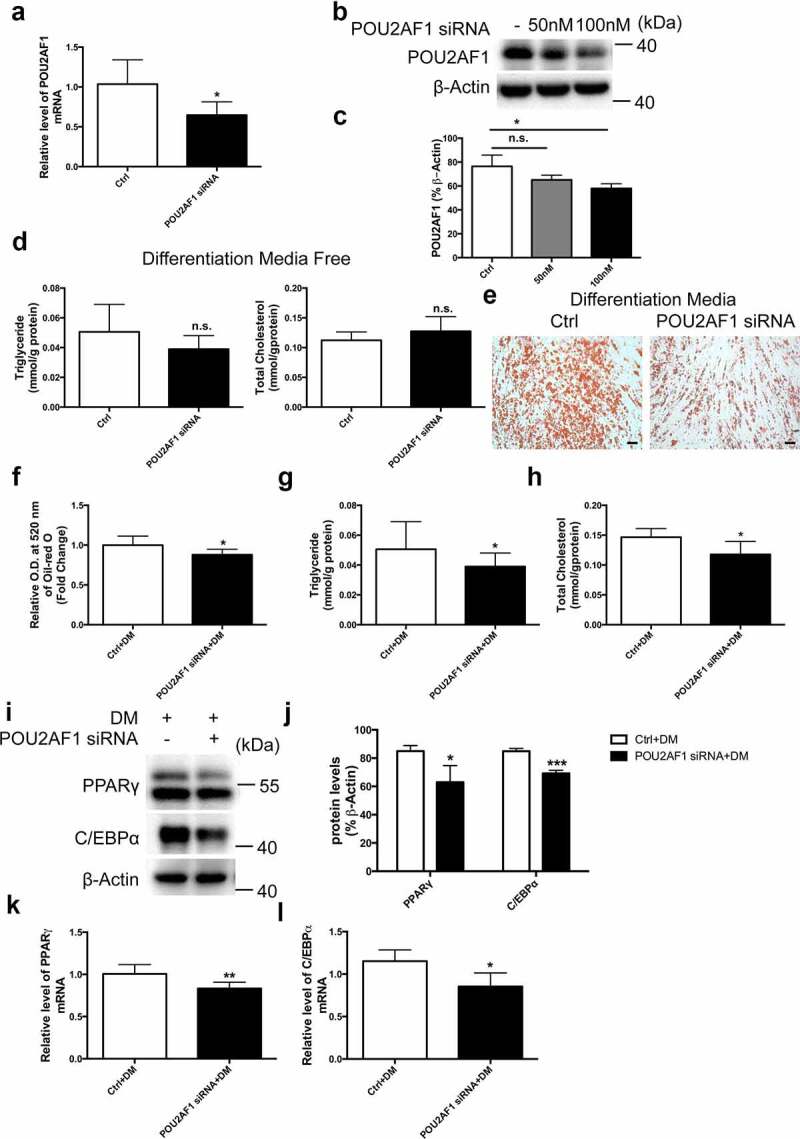
Silencing of POU2AF1 in hMSCs inhibited adipogenic differentiation after adipogenic treatment. (a) Quantification of POU2AF1 levels relative to β-Actin. (b) Expression of POU2AF1 was assessed by western blot. (c) Quantification of POU2AF1 levels relative to β-Actin. (d) Triglyceride and total cholesterol content were determined in hSMCs without adipogenic treatment. (e) Neutral lipids formation was determined by oil-red O staining. (f) Oil-red O extracted with isopropanol was measured at O.D. 520 nm. Triglyceride (g) and total cholesterol content (h) were determined in hSMCs after adipogenic treatment. (i) Expression of PPARγ and C/EBPα were assessed by western blot. (j) Quantification of indicated protein levels relative to β-Actin. (k) Quantification of PPARγ levels relative to β-Actin. (l) Quantification of C/EBPα levels relative to β-Actin. (Scale bar: 50 μm. n = 3 for each experiment. *P < 0.05, **P < 0.01, ***P < 0.001)

Transfection of the siRNA that targeted POU2AF1 substantially down-regulated endogenos POU2AF1 mRNA and protein levels (Figure 4a-c). The effect was dose-dependent and 100 nM of POU2AF1 siRNA was used throughout the study (Figure 4b-c). The measurement of triglyceride and total cholesterol demonstrated that POU2AF1 knockdown did not significantly alter hMSCs spontaneous adipogenic differentiation (Figure 4d). However, transfection of POU2AF1 siRNA significantly decreased the number of differentiated adipocytes after adipogenic treatment (Figure 4e-f). The measurement of triglyceride and total cholesterol also indicated that POU2AF1 siRNA inhibited hMSCs adipogenic differentiation after adipogenic treatment (Figure 4g-h). Expression levels of the adipogenic factors were consistently down-regulated after the silencing of POU2AF1. In brief, protein and mRNA levels of PPARγand C/EBPα were decreased after adipogenic treatment (Figure 4i-l).

### HDAC1 was downstream effector of POU2AF1 mediated adipogenic differentiation

**Figure 5.**
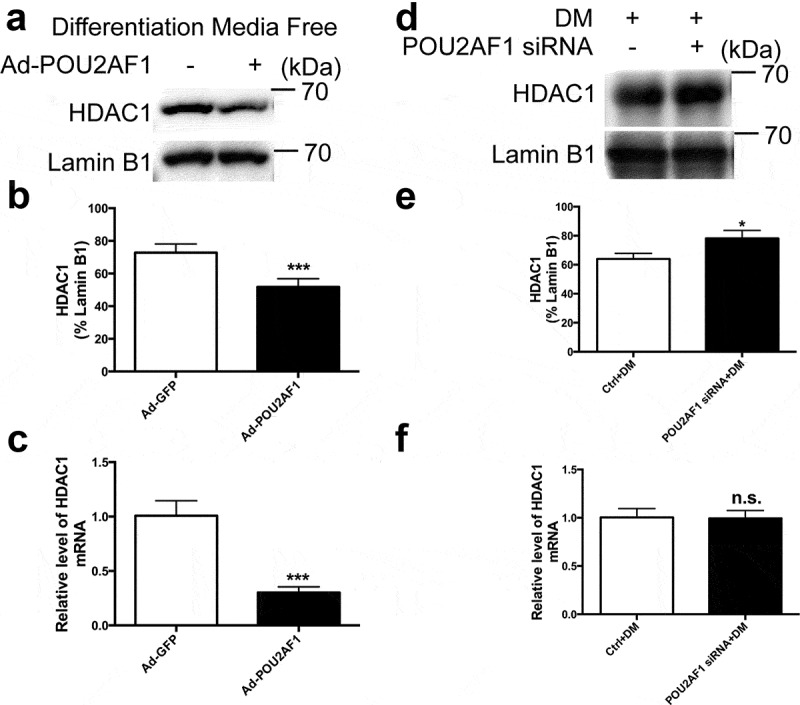
POU2AF1 regulated adipogenic differentiation by HDAC1 signalling. (a) Nuclear protein levels of HDAC1 in hMSCs without adipogenic treatment was assessed by western blot. (b) HDAC1 protein levels were normalized to Lamin B1. (c) Quantification of HDAC1 levels relative to β-Actin. (d) Nuclear protein levels of HDAC1 in hMSCs after adipogenic treatment was assessed by western blot. (e) HDAC1 protein levels were normalized to Lamin B1. (f) Quantification of HDAC1 levels relative to β-Actin. (n = 3 for each experiment. n.s. *P* > 0.05, *P < 0.05, ***P < 0.001)

It has been reported that HDAC1 bound to the C/EBPα promoter in the absence of adipogenic stimuli to block the activation of C/EBPα [13]. To explore whether HDAC1 is the downstream effector in POU2AF1 regulated adipogenic differentiation, we detected the expression of HDAC1 in hMSCs with or without differentiation media. Results demonstrated that overexpression of POU2AF1 alleviated the translocation of HDAC1 to the nucleus (Figure 5a-b). Consistently, the mRNA level of HDAC1 was also reduced (Figure 5c).

To confirm whether HDAC1 is involved in POU2AF1 regulated adipogenic differentiation, we measured HDAC1 in POU2AF1 knockdown hSMCs after adipogenic treatment. Western blot analysis revealed that the protein level of HDAC1 was upregulated (Figure 5d-e), but the mRNA level of HDAC1 was not altered (figure 5f).

**Figure 6.**
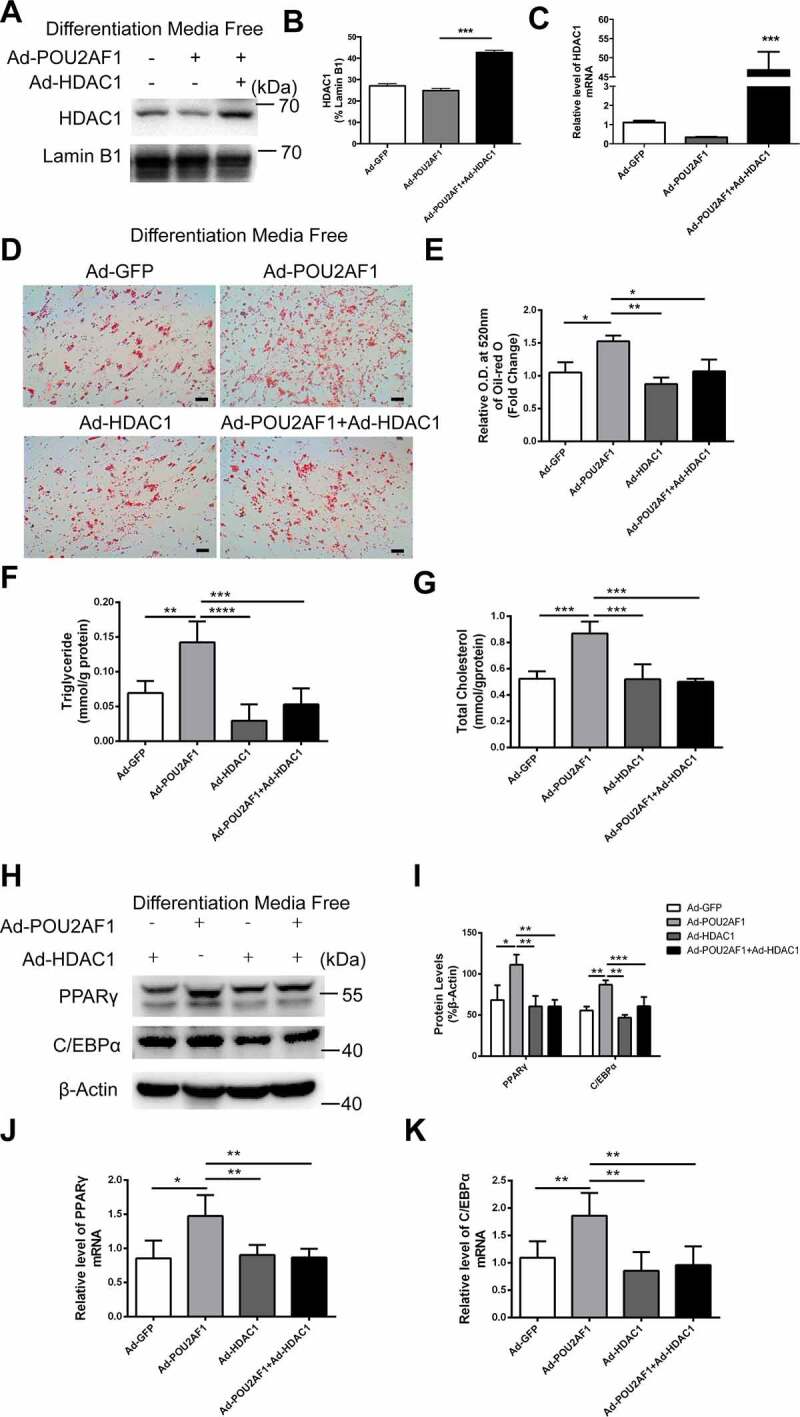
Overexpression of HDAC1 abolished the promotion effect of POU2AF1 on spontaneous adipogenic differentiation. Overexpression of HDAC1 was verified by western blot (a-b) and real-time PCR (c). (d) Neutral lipids formation was determined by oil-red O staining. (e) Oil-red O extracted with isopropanol was measured at O.D. 520 nm. Triglyceride (f) and total cholesterol content (g) were determined in hSMCs without adipogenic treatment. (h) Expression of PPARγ and C/EBPα were assessed by western blot. (i) Quantification of indicated protein levels relative to β-Actin. (j) Quantification of PPARγ levels relative to β-Actin. (k) Quantification of C/EBPα levels relative to β-Actin. (Scale bar: 50 μm. n = 3 for each experiment. *P < 0.05, **P < 0.01, ***P < 0.001)

To further determine whether HDAC1 is the main protein mediating POU2AF1 regulated adipogenic differentiation, we co-transfected Ad-POU2AF1 and Ad-HDAC1, comparing with transferring Ad-HDAC1 only. Overexpression of HDAC1 was verified with western blot and real-time PCR analysis (Figure 6a-c). Co-transfection of Ad-POU2AF1 and Ad-HDAC1 partially reversed the promotion effect of POU2AF1 overexpression in hASMCs spontaneous adipogenic differentiation (Figure 6d-g). Results revealed that Co-transfection of Ad-POU2AF1 and Ad-HDAC1 also reversed the protein and mRNA levels of PPARγ and C/EBPα, especially C/EBPα (Figure 6h-k). These data suggest that HDAC1 is the downstream effector in POU2AF1 regulated adipogenic differentiation, while simple overexpression of HDAC1 had no such effect.

## Discussions

In the present study, we found that POU2AF1 was upregulated in overweight people and POU2AF1 was increased during adipocyte differentiation. Overexpressing POU2AF1 promoted spontaneous hAMSCs adipogenesis and silencing POU2AF1 inhibited hAMSCs adipogenesis. HDAC1 signalling was a potential key mechanism in mediating the promotion effects of POU2AF1.

Body mass index of 30 kg/m^2^ or higher is defined as obesity which is the result of complex relationship between genetic, socioeconomic and cultural influences. For adults, a BMI of 25.0 to 29.9 kg/m^2^ is defined as overweight [21]. Worldwide, the prevalence rate for being overweight or obese between 1980 and 2013 increased 27.5% for adults and 47.1% for children, for a total of 2.1 billion individuals considered overweight or obese [22]. Obesity is the result of overweight progressing to a certain stage. If we can effectively control or reverse the accumulation of adipose during the overweight period, the incidence of obesity will be greatly reduced.

There are many reasons of being overweight, and the most common mechanism of adipose tissue expansion involves hypertrophy and hyperplasia. Abdominal adipose excessive accumulation is more closely associated with the risk of disease than excess total body fatness per se [23,24]. As for the two ways of adipose tissue increases, hypertrophy is the way adipose tissue expands from existing adipocytes. Hyperplasia is the way resident tissue precursors differentiates into new adipocytes [7].

In the present study, we focused on hyperplasia. All adipocytes differentiate from MSCs. Considering that visceral fat plays a more important role in obesity complications, this study investigated MSCs from the retroperitoneal adipose tissue. Although we didn’t count the cell number during differentiation, the changes of expressions of RNA and protein levels and downstream products of lipid formation firmly indicated the procedure of hyperplasia. We explored the effects of POU2AF1 on spontaneous hMSCs adipogenesis without adipogenic treatment and the effects of POU2AF1 on hMSCs adipogenesis with adipogenic treatment. To our best knowledge, this is the first report on promotion adipogenesis function of POU2AF1.

POU2AF1 has been previously reported as a B-cell-specific transcriptional co-activator protein to regulate B-cell development and function [18]. Known diseases associated with POU2AF1 include primary biliary cholangitis, Hodgkin’s lymphoma, and lymphocytic depletion. POU2AF1 impairs B cell differentiation at the earliest stage of development [25]. As POU2AF1 is widely expressed in many kinds of human cells, with the combination of our experiments that it involved in natural MSCs differentiation, we make assumptions that it may promote adipocyte differentiation and thus promote lipid droplets. This could be the mechanism of developing overweight by increasing VF via POU2AF1. POU2AF1 usually binding with Oct-1 and Oct-2 proteins to regulate gene expression. Whether POU2AF1 is promoting adipogenesis through Oct-1/Oct-2/POU2AF1 tri-complex needs further verification.

PPAR-γ is indispensable for adipocyte differentiation in vivo and in vitro. PPAR-γ has become a major target for the treatment of type 2 diabetes mellitus. Accordingly, the prescription of PPAR-γ inhibitors such as thiazolidinediones were widely used. C/EBP-α is one of the most important downstream effects of PPAR-γ. Overexpression C/EBP-α in cultured fibroblasts is sufficient to drive adipogenesis and this effect requires PPAR-γ. PPAR-γ also requires C/EBP family proteins to activate transcription of genes expressed in mature adipocytes [26]. Therefore, we selected markers of these two classical lipid pathways to verify the regulation of POU2AF1.

Inhibiting class I histone deacetylases (HDACs) reduces adiposity, increases energy expenditure, and improves insulin sensitivity in obese mice. Fenfen Li [27] has demonstrated that HDAC1 is down-regulated during brown adipocyte differentiation. A dissociation of HDAC1 from promoters of mouse brown fat (BAT)-specific genes may lead to adrenergic activation induced BAT-specific gene expression in brown adipocytes. Overexpression of HDAC1 in BAT brown adipocytes suppressed brown adipocyte-specific gene expression, including C/EBP. Glucocorticoids have been reported to regulate C/EBP-β by directing the acetylation of C/EBP-β and mediating the interaction of C/EBP-β and HDAC1 [28]. It has also been reported that HDAC1 binds to the C/EBP-α promoter in undifferentiated MSCs to induce adipogenic differentiation of MSCs [16]. Although it has been rarely reported the direct interaction between POU2AF1 and HDAC1 at present, based on the existing research, we think that HDAC1 may act similarly banding to POU2AF1 leading to reduced differentiation of MSCs and POU2AF1 possibly mediate adipogenesis via HDAC1 signalling, which needs to be verified by CO-IP assay through further experiments.

Several limitations in our study must be acknowledged. Firstly, adipose cells were taken from cancer patients, not normal people. Subject to ethics, we could not obtain visceral white adipose from normal people through the invasive pathway. Secondly, co-immunoprecipitation should be necessary to confirm the relationship between POU2AF1 and HDAC1. What’s more, animal models should be established to demonstrate the relationship between the expression of POU2AF1 and adipogenic differentiation as well as further relative disease.

## Conclusion

Taken together, we present for the first time the role of POU2AF1 in adipocyte differentiation and the possible signalling between POU2AF1 and HDAC1. They may be the new targets for inhibiting adipose production and accumulation and it could provide new opportunities for future prevention, control, and even a cure for overweight, obesity and related diseases. Such effects have the potential to reduce cardiovascular events, although further clinical trials are required to determine this possibility.

## Supplementary Material

Supplemental Material
