# Supplementary material for: POU2AF1 promotes MSCs adipogenesis by inhibiting HDAC1 expression
Source: Adipocyte. 2021 May 5;10(1):251–63. doi: 10.1080/21623945.2021.1918863 (PMC8115553; doi:10.1080/21623945.2021.1918863)
Supplement: Supplemental Material [file KADI_A_1918863_SM1913.zip › Document.rtf]

Supplemental Figure I (A) Cell morphology of hMSCs . (B) Cell viability of hMSCs infected with Ad-GFP or Ad-POU2AF1 assessed by cell counting kit .Triglyceride (C) and total cholesterol content (D) were determined in hMSCs infected with Ad-GFP or Ad-POU2AF1 in the presence or absence of differentiation media. (E) Expression of PPARã and C/EBPá were assessed by western blot. (F) Quantification of indicated protein levels relative to â-Actin. (n=3 for each experiment. n.s. P > 0.05,*P < 0.05)

Supplemental Figure II (A-F) mRNA expression levels of PPARã and C/EBPá in hMSCs infected with Ad-GFP or Ad-POU2AF1 following with or without treatment of differentiation media for 0, 3, 5, 7 days. (n=3 for each experiment. n.s. P > 0.05,*P < 0.05,***P < 0.001)
