# Supplementary figures and images for: POU2AF1 promotes MSCs adipogenesis by inhibiting HDAC1 expression
Source: Adipocyte. 2021 May 5;10(1):251–63. doi: 10.1080/21623945.2021.1918863 (PMC8115553; doi:10.1080/21623945.2021.1918863)

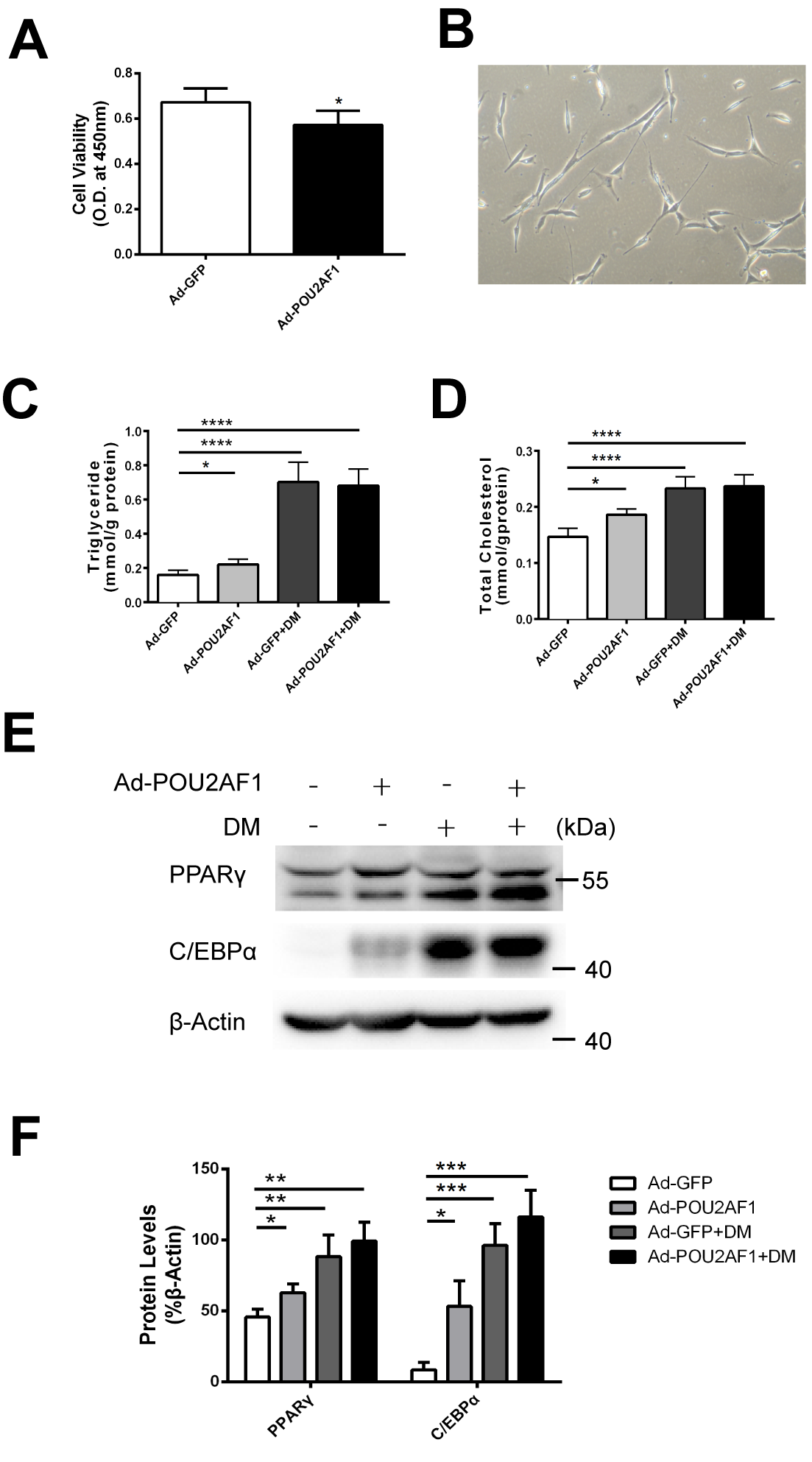

Supplement: Supplemental Material [file KADI_A_1918863_SM1913.zip › S1.tif]

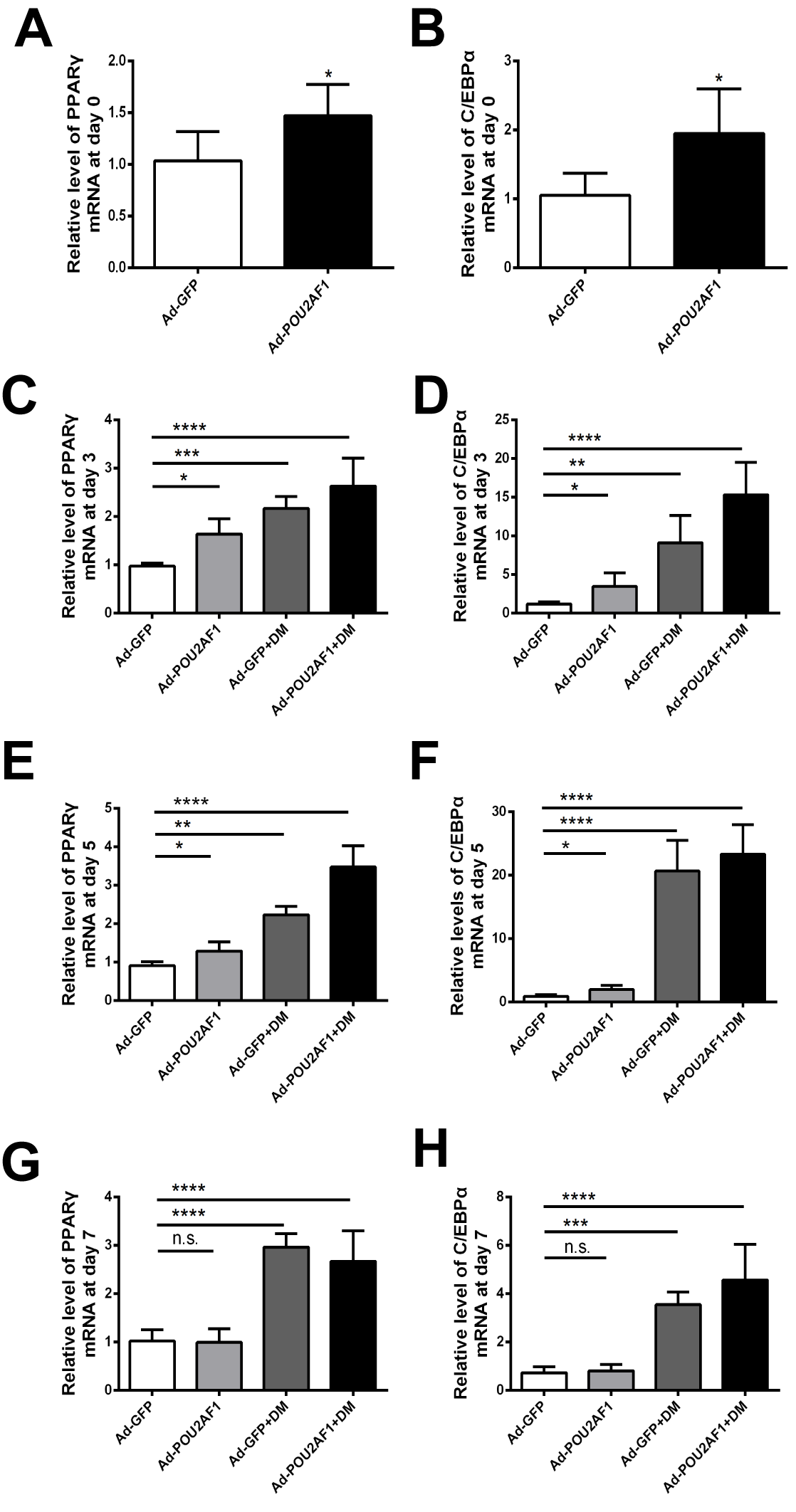

Supplement: Supplemental Material [file KADI_A_1918863_SM1913.zip › S2.tif]
